# Editorial: Enhancing CAR T-cell therapy with imaging

**DOI:** 10.3389/fimmu.2025.1705002

**Published:** 2025-12-09

**Authors:** Egesta Lopci, Dorine de Jong, Laurent Dercle

**Affiliations:** 1 Nuclear Medicine, IRCCS - Humanitas Research Hospital, Rozzano, Italy; 2 RefleXion Medical, Hayward, CA, United States; 3 Department of Radiology, New York-Presbyterian/Columbia University Irving Medical Center, New York, NY, United States

**Keywords:** multimodal imaging, MRI, PET, CAR-T, optical imaging, cell tracking

Chimeric Antigen Receptor T-cell (CAR-T) therapy has revolutionized cancer treatment, particularly for hematological malignancies, by genetically engineering patient-derived T cells to specifically recognize and attack cancer cells (1–3). Despite its success in blood cancers, extending CAR-T therapy to solid tumors has been hindered by various biological barriers, including poor infiltration of CAR-T cells into tumor sites and an immunosuppressive tumor microenvironment that reduces CAR-T cell persistence and activity (3–7). Understanding the dynamic behavior of CAR-T cells in solid tumors, such as their trafficking patterns, localization, and functional status, remains crucial for improving therapeutic outcomes.

Advanced molecular and cellular imaging techniques are essential tools for the real-time, non-invasive monitoring of CAR-T cells within living organisms. These technologies, which include bioluminescence imaging (BLI), fluorescence imaging, magnetic resonance imaging (MRI), positron emission tomography (PET), and photoacoustic imaging, allow researchers to track CAR-T cell distribution, expansion, and tumor infiltration over time (8). Each imaging modality offers unique advantages, with PET providing high sensitivity and whole-body tracking, MRI delivering detailed anatomical resolution, and optical methods enabling multiparametric assessments. Collectively, these modalities offer comprehensive insights into the *in vivo* kinetics and therapeutic effects of CAR-T cells (3–8).

This Research Topic, recently compiled for Frontiers in Immunology, aims to decipher the role of imaging techniques in the context of CAR-T therapy by enhancing the assessment of CAR-T cell biodistribution, trafficking kinetics, targeting, and monitoring within solid tumors. The contributions published have highlighted some of the aspects that most importantly relate to chimeric antigen technology. Gehrke et al. first illustrated the utility of direct CAR-T cell visualization by means of dSTORM super-resolution microscopy, which allowed the research team to detect the surface expression of CARs targeting SLAMF7, BCMA, and CD19 with minimal background. The authors were also able to determine T cell subtype, donor material, and CAR construct as contributing factors shaping CAR surface expression and to identify the putative influence of CAR surface expression on the activation state of CAR-T cells. They concluded that their work could potentially be a basis for more intricate and combinatorial studies to further improve the efficacy of CAR-T cell immunotherapy, predict therapeutic outcomes, and ensure optimal patient care.

On the other hand, the clinical impact of metabolic imaging was discussed in the article submitted by Ladbury et al., who used F18-fluorodeoxyglucose positron emission tomography (PET) after bridging radiation therapy (bRT) to predict the prognosis of B-cell lymphoma patients undergoing CAR-T cell therapy. The analyzed parameters included metabolic tumor volume (MTV), maximum standardized uptake value (SUVmax), SUVmean, and total lesion glycolysis (TLG). As expected, bRT led to substantial reductions in all of these parameters, with the extent of delta variation significantly correlated with progression-free survival (PFS), freedom from distant progression (FFDP), and local control (LC). The authors concluded that prospective cohort studies are needed to validate the value of interim PET following bRT for quantifying changes in disease burden and associated prognosis.

Another interesting aspect related to immunotherapy in general is the occurrence of atypical responses, and particularly of pseudoprogression (3, 9). For this purpose, Zhao et al. described for the first time a case of pseudoprogression after CAR-T cell therapy in solid tumors. They described the case of an elderly patient with advanced gastric cancer and hepatic metastases who showed enlargement 1 month after CAR-T cell infusion. The lesions were reported to shrink the following month, as confirmed by computed tomography scanning, exhibiting the characteristic behavior of pseudoprogression.

Integrating imaging modalities into CAR-T cell therapy research not only aids in optimizing treatment strategies but also accelerates clinical translation. Imaging facilitates the evaluation of CAR-T cell biodistribution, helps identify barriers such as off-target accumulation or insufficient tumor penetration, and can monitor treatment-related toxicities. Furthermore, engineering CAR-T cells with imaging reporter genes enhances the capability for longitudinal tracking, enabling the assessment of therapeutic efficacy and safety in preclinical models and clinical trials (10). Thus, imaging-driven approaches promise to overcome existing challenges in solid tumor CAR-T cell therapy by offering precise, personalized guidance for cell therapy optimization (6, 7).
